# Same-Day Diagnostic and Surveillance Data for Tuberculosis via Whole-Genome Sequencing of Direct Respiratory Samples

**DOI:** 10.1128/JCM.02483-16

**Published:** 2017-04-25

**Authors:** Antonina A. Votintseva, Phelim Bradley, Louise Pankhurst, Carlos del Ojo Elias, Matthew Loose, Kayzad Nilgiriwala, Anirvan Chatterjee, E. Grace Smith, Nicolas Sanderson, Timothy M. Walker, Marcus R. Morgan, David H. Wyllie, A. Sarah Walker, Tim E. A. Peto, Derrick W. Crook, Zamin Iqbal

**Affiliations:** a Nuffield Department of Clinical Medicine, University of Oxford, John Radcliffe Hospital, Oxford, United Kingdom; b Wellcome Trust Centre for Human Genetics, University of Oxford, Oxford, United Kingdom; c School of Life Sciences, University of Nottingham, Nottingham, United Kingdom; d Foundation for Medical Research, Mumbai, India; e Regional Centre for Mycobacteriology, PHE Public Health Laboratory, Birmingham Heartlands Hospital, Birmingham, United Kingdom; f Public Health England, Wellington House, Lambeth, London, United Kingdom; g Microbiology Laboratory, John Radcliffe Hospital, Oxford University Hospitals NHS Trust, Oxford, United Kingdom; h The Jenner Institute, University of Oxford, Oxford, United Kingdom; i National Institute for Health Research (NIHR) Oxford Biomedical Research Centre, John Radcliffe Hospital, Oxford, United Kingdom; Memorial Sloan-Kettering Cancer Center

**Keywords:** DNA sequencing, Mycobacterium tuberculosis, antibiotic resistance, diagnostics

## Abstract

Routine full characterization of Mycobacterium tuberculosis is culture based, taking many weeks. Whole-genome sequencing (WGS) can generate antibiotic susceptibility profiles to inform treatment, augmented with strain information for global surveillance; such data could be transformative if provided at or near the point of care. We demonstrate a low-cost method of DNA extraction directly from patient samples for M. tuberculosis WGS. We initially evaluated the method by using the Illumina MiSeq sequencer (40 smear-positive respiratory samples obtained after routine clinical testing and 27 matched liquid cultures). M. tuberculosis was identified in all 39 samples from which DNA was successfully extracted. Sufficient data for antibiotic susceptibility prediction were obtained from 24 (62%) samples; all results were concordant with reference laboratory phenotypes. Phylogenetic placement was concordant between direct and cultured samples. With Illumina MiSeq/MiniSeq, the workflow from patient sample to results can be completed in 44/16 h at a reagent cost of £96/£198 per sample. We then employed a nonspecific PCR-based library preparation method for sequencing on an Oxford Nanopore Technologies MinION sequencer. We applied this to cultured Mycobacterium bovis strain BCG DNA and to combined culture-negative sputum DNA and BCG DNA. For flow cell version R9.4, the estimated turnaround time from patient to identification of BCG, detection of pyrazinamide resistance, and phylogenetic placement was 7.5 h, with full susceptibility results 5 h later. Antibiotic susceptibility predictions were fully concordant. A critical advantage of MinION is the ability to continue sequencing until sufficient coverage is obtained, providing a potential solution to the problem of variable amounts of M. tuberculosis DNA in direct samples.

## INTRODUCTION

The long-standing gold standard for Mycobacterium tuberculosis drug susceptibility testing (DST) is the phenotypic culture-based approach, which is time-consuming and laborious. First-line tuberculosis (TB) treatment includes four drugs (rifampin, isoniazid, ethambutol, and pyrazinamide), but with the spread of multidrug-resistant strains, there is a growing need for data on second-line drugs, including the fluoroquinolones and aminoglycosides.

Because of the long turnaround times for phenotypic testing (up to 2 months), it is often preceded by WHO-endorsed molecular methods such as the GenoType MTBDRplus and MTBDRsl assays (Hain Lifescience GmbH, Germany) and Xpert MTB/RIF (Cepheid, USA). These potentially culture-free, PCR-based tests rapidly identify isolates to the species level and detect the most common drug resistance-conferring mutations. However, this technology is limited by the number of mutations that can be probed. This limitation is of concern, given the many low-frequency drug resistance-conferring mutations in M. tuberculosis, particularly for second-line drugs (1). Consistent with this concern, the proportion of phenotypically resistant samples that are detectable by MTBDRplus ranges from 21 to 25% for the second-line drugs capreomycin and kanamycin (2) to 98.4% and 91.4% for the critical first-line drugs rifampin and isoniazid (3). A potential solution is to sequence amplicons targeting a wider range of resistance-conferring genes, as previously demonstrated (4).

The potential of whole-genome sequencing (WGS) as a diagnostic assay has been repeatedly identified (5–7). Recent studies based on WGS of mycobacteria have evaluated WGS-based susceptibility predictions (1, 8–10), species identification, and elucidation of epidemiology (11–16). This has culminated in the first successful application of WGS as a clinical diagnostic for mycobacteria from early positive liquid cultures (16). Moreover, WGS was performed at a cost comparable to that of existing phenotypic assays and offered shorter turnaround times.

Generating WGS information directly from patient samples and avoiding the need for culture would be transformative. However, direct samples contain highly variable amounts of mycobacterial cells mixed with other bacterial and human cells, with the latter accounting for up to 99.9% of the DNA present. Furthermore, mycobacterial cells may aggregate because of the high mucus content of some samples, meaning that the sample volume and acid-fast bacillus (AFB) count may not represent the total quantity of mycobacteria available. Direct samples therefore require preprocessing to homogenize and enrich for mycobacteria by depleting other cells/DNA. The challenges of direct sample processing were illustrated by two studies assessing the feasibility of direct WGS of clinical samples (17, 18). By sequencing eight smear-positive sputum samples subjected to differential lysis followed by DNA extraction with a commercial kit, Doughty and colleagues were able to achieve only a 0.002× to 0.7× depth of coverage for M. tuberculosis, with 20.3 to 99.3% of the sequences mapping to the human genome. Seven of eight samples could be assigned to the M. tuberculosis complex, but none had sufficient data for drug susceptibility prediction. In a second study, Brown and colleagues applied a SureSelect target enrichment method (Agilent, USA) to capture M. tuberculosis DNA prior to WGS. Twenty of 24 smear-positive samples achieved 90% genome coverage with ≥20× depth, sufficient for prediction of species and antibiotic susceptibility. However, the protocol was slow (2 to 3 days) and may be prohibitively expensive for use in low-income settings.

In this study, we tested a simple, low-cost DNA extraction method using Illumina MiSeq for WGS of 40 smear-positive, primary respiratory samples from M. tuberculosis-infected patients. We evaluated the protocol in terms of the DNA obtained, the species assignment of the sequenced reads, and our ability to obtain key clinical data (detection of M. tuberculosis and antibiotic susceptibility prediction) along with epidemiological information (placement on a phylogenetic tree). These data would enable a single test to deliver the core information for both patient and public health in <48 h by using Illumina-based WGS. We also developed an approach for WGS by using the highly portable, random-access, Oxford Nanopore Technologies (ONT) MinION, reducing the potential turnaround time to <12 h.

## RESULTS

### DNA extraction protocol and evaluation of Illumina sequencing output.

DNA was extracted from 40 Ziehl-Neelsen (ZN)-positive direct respiratory samples, of which 38 were culture confirmed M. tuberculosis positive (culture positive) and 2 were culture negative. DNA was also extracted from 28 available corresponding mycobacterial growth indicator tube (MGIT) cultures. All direct samples were the remainders of specimens available after processing by the routine laboratory and therefore had various volumes (median, 1.5 ml; interquartile range [IQR], 0.5 to 3.1 ml; range, 0.25 to 15 ml) and age (median, 30 days from collection to processing; IQR, 15 to 45 days; range, 0 to 67 days). Most direct samples (78%; 31/40) could therefore be considered suboptimal on the basis of a low volume (≤1 ml), a long storage time (≥30 days), or both.

**FIG 1:**
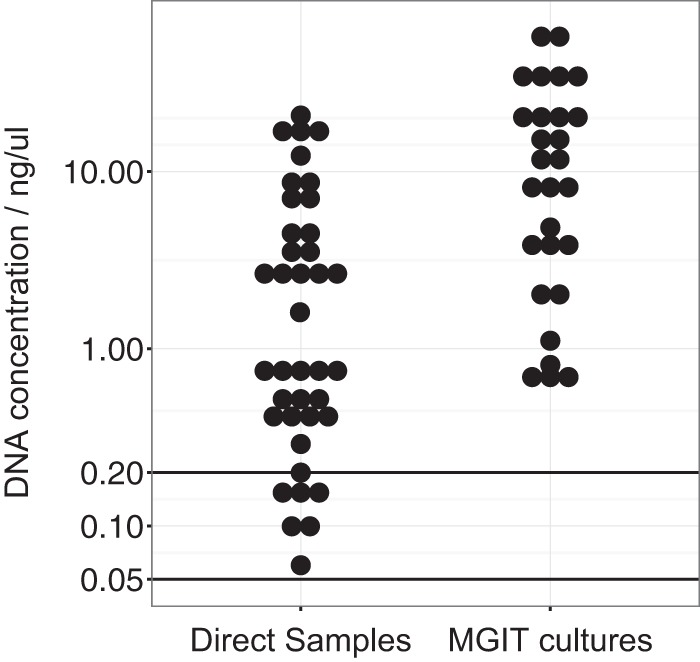
Concentrations of DNA extracted from MGIT cultures and direct clinical samples. Each dot represents a single extraction. The horizontal line at 0.2 ng/μl represents the DNA concentration theoretically required for MiSeq library preparation. The horizontal line at 0.05 ng/μl represents the minimum DNA concentration used for MiSeq library preparation from direct samples in this study. One sample not shown as DNA was below the limit of detection.

After DNA extraction, 33/40 (83%) direct samples and all 28 MGIT cultures yielded ≥0.2 ng/μl DNA, the amount recommended for MiSeq Illumina library preparation (Fig. 1).

There was no evidence that the DNA yield was affected (in either multivariable or univariable models) by (i) the sample type (sputum or bronchoalveolar lavage fluid) (*P* = 0.94; univariable linear regression), (ii) AFB scoring (from +1 to +3) (*P* = 0.56), (iii) the storage time prior to DNA extraction (days from collection) (*P* = 0.51), or (iv) the sample volume (*P* = 0.28). Although the DNA concentration was measured and recorded after extraction, further data on DNA quality (e.g., the DNA integrity number [DIN] provided by TapeStation [Agilent, USA]) were not routinely recorded.

In total, 39/40 direct samples with detectable DNA (37 culture positive, 2 culture negative) and 27/28 MGIT cultures were sequenced with Illumina MiSeq. One MGIT culture was not sequenced because the corresponding direct sample failed to yield measurable DNA. We used a lower-than-recommended DNA concentration threshold for MiSeq library preparation (>0.05 ng/μl rather than >0.2 ng/μl) on the basis of previous experience with the sequencing of mycobacterial cultures with suboptimal amounts of DNA (19). Six (15%) of 40 samples yielded DNA below the 0.2-ng/μl threshold. All sequenced direct samples produced ≥1.5 million reads (median, 3.6 million; IQR, 2.9 to 5.0 million; range, 1.5 to 12 million), as did all MGIT cultures (median, 3.1 million; IQR, 2.8 to 3.3 million; range, 2.0 to 4.1 million).

### Contamination levels of direct and MGIT samples.

**FIG 2:**
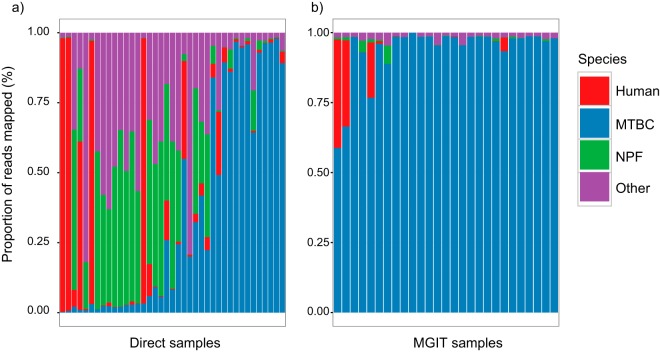
Proportions of reads assigned to various species categories in each sample sorted by increasing total counts of M. tuberculosis complex (MTBC) reads. (a) Direct samples show that removal of human DNA (red) has been broadly successful, but removal of NPF (green) and other bacteria (purple) had more variable success. (b) MGIT samples show much more uniform dominance of M. tuberculosis reads, as expected after 2 weeks of culture designed to favor mycobacterial growth.

We assigned reads to the categories M. tuberculosis, human, nasopharyngeal flora (NPF), and other by mapping (see Materials and Methods). Seventy-seven percent (30/39) of the direct samples contained <10% human reads. However, only 46% (18/39) contained <10% NPF and other bacterial reads, and 26% (10/39) contained >40% reads from nonmycobacterial, non-NPF, bacteria (Fig. 2a). In comparison, MGIT culture samples showed much less contamination, as expected (Fig. 2b).

### Recovery of M. tuberculosis genome.

**FIG 3:**
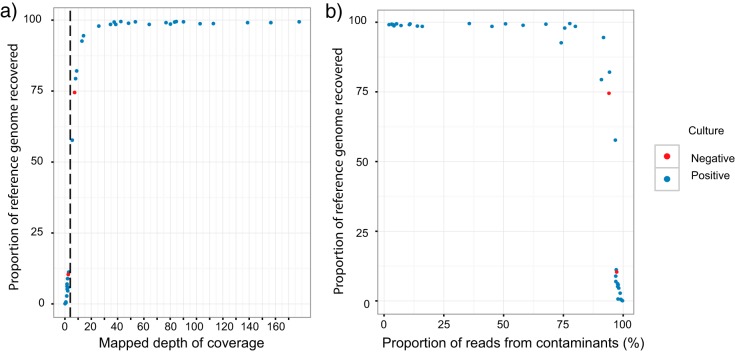
Recovery of M. tuberculosis genome in direct samples and robustness to contamination. (a) Depth versus proportion of the M. tuberculosis reference recovered (at >5× depth). The vertical dotted line at 3× depth is the threshold used for resistance prediction in this study. (b) Proportion of contamination (reads not mapping to M. tuberculosis reference) versus proportion of genome recovered. Samples with <95% of the M. tuberculosis genome recovered all have >75% contaminated reads.

Figure 3a shows the distribution of the M. tuberculosis reference genome depth of coverage across direct samples. Twenty-one of 39 samples have >12× depth and recover >90% of the genome, and 14/39 samples have <3× depth and recover <12% of the genome. The vertical dotted line delineates our threshold of 3× coverage, below which resistance predictions were not made. Figure 3b shows the amount of contamination (reads not mapping to M. tuberculosis) per sample. Ten samples had <15% contaminant reads, although contamination levels increased as high as 75% before the proportion of the M. tuberculosis genome recovered started to drop. Low numbers of M. tuberculosis reads could also reflect poor DNA quality from samples stored for long periods, as most of the samples with <80% reference genome coverage (12/17, 71%) were >3 weeks old before extraction.

### Concordance of results from direct and MGIT samples.

**FIG 4:**
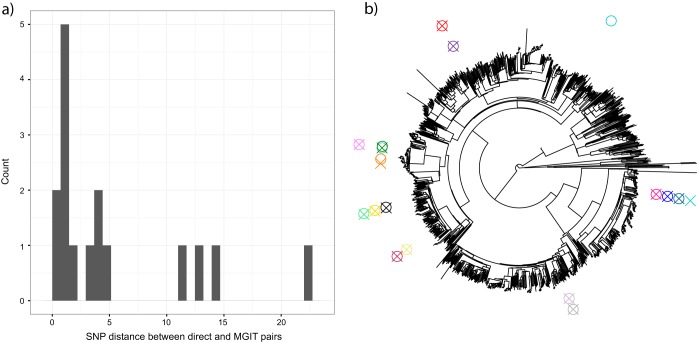
Genotypic concordance between direct and paired MGIT samples. (a) Histogram of genetic (SNP) differences, excluding the one pair that differ by 1,106 SNPs; the median (and modal) difference is 1, and thus, direct sequencing is identifying the same strain of M. tuberculosis that culture-based sequencing would. (b) Placing direct/MGIT pairs on a phylogenetic tree of 3,480 samples shows the distribution of samples across world diversity. A circle indicates the sequence from the direct sample, and an X indicates the sequence from the corresponding MGIT sample. For the 1 pair (of 17) with 1,106 differences (turquoise), the MGIT sample is placed very close to the other samples (zero SNP differences from one [MGIT] sample and five SNP differences from the others) and so is possibly due to a labeling error.

We took a set of 68,695 high quality single-nucleotide polymorphisms (SNPs) obtained from an analysis of 3,480 samples (1) and genotyped all of the samples at these positions (see Materials and Methods). This allowed us to calculate the genetic distance between the 17 paired MGIT and direct samples (after excluding 10 pairs where the direct sample had <5× coverage, to avoid systematic undercalling in direct samples). The median (and modal) SNP difference was 1 (Fig. 4a), with one outlier pair of samples that differed by 1,106 SNPS (discussed below), and all other differences were ≤22 SNPs.

We placed 17 paired direct and MGIT samples on the phylogenetic tree from reference 1 (see Materials and Methods). Our samples were distributed across global diversity (Fig. 4b; tree thinned to aid visibility). For the pair with 1,106 SNP differences, the direct sample was isolated on the tree but the MGIT sample was placed very closely to three other pairs (0 SNP difference from one MGIT sample, and 5 SNP differences from the others). Although this might result from different strains being present within the host, a within-laboratory labeling error or cross-contamination is also possible.

### No evidence of higher diversity in direct samples.

Comparing direct/MGIT pairs where both samples had at least a 20× mean depth of coverage on the M. tuberculosis reference, the median number of high-confidence (see Materials and Methods) heterozygous sites was 25 in both direct and MGIT samples. There was no clear trend of greater genome-wide diversity in direct samples (see Fig. S1 in the supplemental material).

### Detection of M. tuberculosis in culture positive/negative samples.

All sequenced culture-positive (37/39) direct M. tuberculosis samples were successfully identified by Mykrobe predictor to complex level (37/37) and 95% to species level (35/37), including 13/37 (35%) where the mean depth of coverage was <3×. All MGIT cultures were identified as M. tuberculosis. We were also able to identify M. tuberculosis in two of two direct samples with low AFB scores (+1) and no growth in MGIT culture; these may represent dead bacilli from a patient undergoing treatment.

### Antibiotic resistance prediction.

In total, 168 predictions for first-line (*n* = 96) and second-line (*n* = 72) antibiotic susceptibility were made for the 24/37 (65%) direct samples that had at least 3× depth (see Tables S1 and S2). For the 13/37 (35%) samples that had <3× depth, no resistance predictions were made. This included one of two culture-negative samples.

Ninety-two (96%) of 96 predictions for the first-line antibiotics were concordant with reference laboratory DST. The four mismatches (three pyrazinamide mixed genotypes with both R and S alleles present and one rifampin-resistant genotype with a sensitive phenotype) were found across three samples, all from the same patient (patient 2 in Table S2), that had a variable phenotype for rifampin and pyrazinamide. The resistant genotype for rifampin was consistent across all three samples from this patient (rpoB_I491F). There is evidence that this mutation causes resistance but that the phenotype is often reported as sensitive (1, 20, 21). The mixed genotype for pyrazinamide was again consistent with the presence of both R and S alleles on pncA_V7L across all three samples, whereas the phenotype varied. This mutation is also known to confer resistance in samples reported as phenotypically sensitive (1). Further, one of three samples from this patient (602112 in Table S2) contained two additional mutations conferring resistance to isoniazid and pyrazinamide, katG_S315T and pncA_T135P, respectively, which were not detected in the previous or following sample. This variation between genotypes from same-patient samples taken over time may represent ongoing evolution, changing population size, and within-patient diversity of M. tuberculosis, as previously demonstrated by Eldholm et al. (22). In addition to the above, WGS provided 72 predictions for second-line antibiotics where DST was not attempted.

The 13/37 samples that yielded insufficient WGS data for resistance prediction had a higher proportion of other bacterial DNA (Fig. 3b, a median of 96% and an IQR of 38 to 70% versus a median of 12% and an IQR of 0 to 67% in those where resistance prediction was possible [rank sum *P* = 0.01]).

### Sub-24-h turnaround time with Illumina MiniSeq.

Illumina MiniSeq sequencing of three samples (single run; one pure BCG DNA, two negative sputum DNA spiked with BCG DNA) was completed in 6 h 40 min. The BCG reference genome coverage was 31 to 33× in spiked samples and 84× in pure BCG DNA (Table 1). In all cases, the species/strain was correctly identified as M. bovis BCG and pyrazinamide resistance was correctly identified because of an H57D mutation in *pncA*.

**TABLE 1:** Yields from pure BCG and negative sputum spiked with BCG sequenced by Illumina MiniSeq

Sample	Estimated no. of fmol loaded	Yield (Mb)	Read length (bp)	Mean BCG coverage depth (no. of reads)
Pure BCG TB1_N716	800	381	101	84.0
50% BCG TB1_N718	800	244	101	31.0
50% BCG TB1_N719	800	257	101	33.0

### Modified method for ONT MinION.

A new PCR-based rapid 1D MinION protocol was tested by using extracted BCG DNA, ZN-negative sputum DNA spiked with BCG DNA, and R9 flow cells (see Materials and Methods). Analysis of genome-wide coverage distribution confirmed that use of PCR had not led to significant coverage bias (see Fig. S2) and that >95% of the reference genome attained coverage >5× for all samples. In all cases, Mykrobe correctly identified the species as M. bovis and the strain as BCG (Table 2). Amplification with Phusion High-Fidelity master mix resulted in the highest yield (760 Mb, with 68× coverage of BCG). All MinION experiments resulted in correct identification of the H57D mutation in *pncA* that confers pyrazinamide resistance in BCG, but in the 5% spike, this call was filtered out as it only had k-mer coverage of 1× on the resistant allele and did not achieve the required confidence threshold. In all cases, no false resistance calls were made, but deep coverage was needed to be able to genotype all 175 mutations in the catalogue (see Table S3). Although the pure BCG/R9 and 15% BCG/Phusion/R9 runs missed only 3/175 and 1/175 mutations, respectively (Table 2), only the R9.4 sequencing run (below) allowed all of the mutations to be typed.

**TABLE 2:** Yields from pure BCG and negative sputum spiked with BCG both sequenced by the MinION 1D protocol

Model	Sample	No. of fmol loaded	Read count	Yield/Mb	Avg read length (kb)	Mean BCG coverage depth (no. of reads)	H57D R allele k-mer coverage depth (no. of k-mers)^*a*^	% of mutations typed (no. not typed)
R9	Pure cultured BCG	NA^*b*^	297,239	360	1.2	80	17	99 (1)
R9	5% BCG LongAmp	82	182,670	559	2.0	19	1^*c*^	47 (93)
R9	10% BCG LongAmp	76	180,507	467	1.8	10	3	56 (77)
R9	15% BCG LongAmp	51	203,285	627	2.0	35	3	90 (18)
R9	15% BCG Phusion	27	184,895	758	2.4	68	10	98 (3)
R9.4	15% BCG Phusion	43	754,338	1,306	1.7	147	16	100 (0)

a k-mer coverage on resistance allele of the H57D mutation in *pncA*, known to be present in BCG.

b NA, data not available.

c Resistance SNP detected but failed confidence threshold and filtered out.

With all five samples sequenced on R9 flow cells, the data yield was highest at the start of the run, with consistent yield profiles. For the Phusion/15% run, we obtained >65% of the data in 8 h and 80% in 10 h (see Fig. S3). Despite the high sequencing error rate (see Fig. S4), high-accuracy genotyping of known SNPs/indels was achievable as described above.

Using two independent methods (see Materials and Methods), we measured a strong bias in the distribution of SNP errors in the consensus of the MinION data. Both methods agreed that the bias was systematic, consistent with a strong A-to-G error bias within a 1D read (see Tables S4 and S5), but differed in their determination of the strength of the bias (28% versus 50% A to G, respectively). A filter to ensure that SNP calls have support from reads mapping to both strands could remove such errors.

### Turnaround time of 12.5 h with ONT R9.4 MinION.

**FIG 5:**
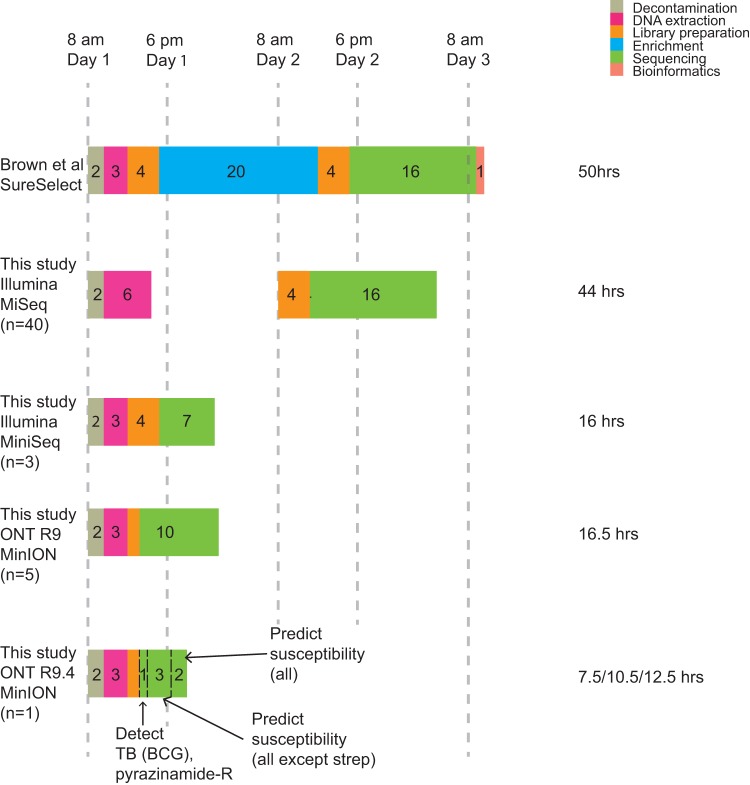
Timelines. We compared the method of Brown et al. with the results of this study obtained with Illumina MiSeq and MiniSeq and ONT MinION. We assumed that no step of the process can be initiated after 6 p.m. or before 8 a.m. The method of Brown et al. has a rapid extraction step but also a 20-h overnight enrichment step, resulting in a 50-h turnaround time. In our study, we did 27-h MiSeq runs (paired 150-bp reads), but since Mykrobe is k-mer based, a 16-h run (paired 75-bp reads) would give equivalent results; we therefore display that potential timeline here. The DNA extraction process was updated for the MiniSeq and MinION experiments, removing the ethanol precipitation step. In normal use, this would take 3 h. The 1.5-h orange rectangle on the MinION time lines includes both PCR and the 10-min sample preparation step. In this experiment, since we used spiked BCG DNA in sputum, we did not use a human depletion step, thus taking only 2 h. This image is intended to show comparable real-use time lines, and so the MiniSeq/MinION time lines are shown with 3-h extraction steps. MiniSeq enables a 16-h turnaround time by sequencing for only 7 h. R9 MinION also delivers sub-24-h results but requires one flow cell per sample. R9.4 MinION gives a 12.5-h turnaround time (6 h of sequencing with real-time [i.e., simultaneous] base calling when used on a single sample).

We sequenced a single sample (15% BCG-spiked, ZN-negative sputum) on the latest R9.4 MinION flow cell (see Materials and Methods). The yield was 1.3 Gb in 48 h. We were able to detect the M. tuberculosis complex, identify the strain as BCG, detect the correct pyrazinamide resistance mutation, and correctly place the sample on the phylogenetic tree after 1 h of sequencing. After 3 h, 170 of 175 mutations were genotyped confidently; after 4 h, we had definitive results for all of the drugs except streptomycin; and after 6 h, we had definitive results for streptomycin and could stop sequencing. One pyrazinamide mutation remained ungenotyped, but since we already had a confident resistance call for pyrazinamide, there would be no need to continue. Sufficient coverage on the final mutation was obtained after a further 3 h (a total of 9 h of sequencing; Table 3). Incorporating 6.5 h for decontamination, DNA extraction, and sample preparation (Fig. 5), this would give a turnaround time of 7.5 h for identification of the species, phylogenetic placement, and initial susceptibility predictions and 12.5 h for complete results.

**TABLE 3:** Susceptibility prediction at time stamps during R9.4 run

Hr	% of AMR mutations typed	No. of mutations ungenotyped (total, 175)	Ungenotyped mutation(s)	Drug(s) awaiting results
1	57.1	75	—^*a*^	All but pyrazinamide
2	88.5	20	*katG* S700, L141, V633, W191, D142, L704; *gid* L26, V41, G34, R47, G117, A205, R118, Q125; *rpoB* H445; *embB* D328, G406; *rpsL* K43; *pncA* T47, K48^*b*^	Isoniazid, streptomycin, rifampin, ethambutol
3	97.1	5	*embB* D328; *gid* G34, A205; *katG* W191; *pncA* T47	Ethambutol, streptomycin, isoniazid
4	98.2	3	*gid* G34, A205; *pncA* T47	Streptomycin
5	98.8	2	*gid* G34; *pncA* T47	Streptomycin
6–9	99.4	1	*pncA* T47	
9	100	0		

a Ungenotyped mutations omitted because 75 is too many to list.

b Further ungenotyped *pncA* mutations could be ignored, as H57D had already been detected at 1 h. The sample was already predicted to be pyrazinamide resistant; thus, pyrazinamide is not listed.

We took our phylogenetic placement of the MiniSeq BCG data as truth, four SNPs distant from a BCG sample on the predefined tree. After 1 h of sequencing with R9.4, we were able to confidently genotype 22,694 of the 68,695 SNPs, placing the sample at the correct leaf of the tree, at an estimated distance of three SNPs. Thus, our genotyping on 1D nanopore reads had, at most, seven errors (three plus four) out of 22,694 SNPs—an error rate below 0.03%.

Finally, on the basis of the performance of the 1.3-Gb R9.4 sequencing run, we estimate (see Materials and Methods) that full susceptibility prediction would fail to be generated for 17 of the 39 sputum samples sequenced here (MiSeq) with <8% M. tuberculosis. However, for the 11/39 samples with >84% M. tuberculosis, species identification and initial susceptibility predictions would be obtained within 20 min of sequencing and full results would be obtained within 93 min (see Fig. S8 and Table S6).

### Costing.

The reagent costs (sample decontamination, extraction, sequencing library preparation, and sequencing) per sample were £96 (MiSeq, 12 samples/run), £198 (MiniSeq, 3 samples/run), £515 (R9 MinION, 1 sample/run), and £101 to £172 (R9.4 MinION, between 3 and 5 samples/run [approximate cost, as a multiplexing kit is not yet available]). See Table S7 for details.

## DISCUSSION

Anticipating a growing knowledge base of the molecular determinants of antibiotic resistance (1), we have developed a method of extracting and purifying mycobacterial DNA from primary clinical samples and producing accurate sequence data in a clinically useful time frame. We have demonstrated first that direct WGS of sputum is possible and gives genotypic DST predictions that are concordant with phenotype and concordant phylogenetic placement with culture-based sequencing. Second, with an Illumina MiSeq sequencer, we can obtain results within 48 h for a <£100 consumable cost per sample. Using Illumina MiniSeq can deliver a same-day test result (16 h) for an estimated consumable cost of £198 per sample. Although the costs presented here only represent reagents, they are still likely to be below those of traditional phenotyping (£518 to provide first- and second-line DST and mycobacterial interspersed repetitive unit-variable number of tandem repeats analysis in a bottom-up costing including, for example, consumables, staff time, and overheads [16] versus £495 for MiSeq under the same costing model). The cost of MiSeq consumables is also well below that of the SureSelect procedure (£203 per sample) (18).

The World Health Organization (WHO) has called for affordable and accessible point-of-care TB diagnostics, including for DST. Current molecular assays provide partial information on some drugs but do not easily scale to incorporate a growing list of recognized resistance mutations. Furthermore, additional assays are currently needed where surveillance or outbreak detection is indicated, at additional cost. A single assay providing diagnostic information and data for surveillance and outbreak detection is therefore an attractive prospect.

In cities where there are large numbers of TB cases (for example, upward of 65,000 TB cases per year in Mumbai), centralized sequencing services taking advantage of high-throughput Illumina sequencing platforms may be applicable. However, at current prices in 2017, the relatively high capital costs and the requirement for a well-equipped laboratory are impediments to implementation across the full range of locations across the world. For a complete solution, the ability to function in various low-tech environments is a practical necessity. The MinION can deliver this, at least on a small scale, as demonstrated in Guinea last year during the Ebola outbreak (23). We confirm here that, despite the high error rate in reads, given deep coverage, it is possible to accurately genotype resistance SNPs by the MinION method applied here. However, widespread implementation would require much larger feasibility studies, similar to those recently conducted to implement MiSeq sequencing from MGIT samples for Mycobacteria diagnosis by Public Health England.

Since with Illumina technology the depth of sequencing is determined in advance (by the number of isolates run in a batch), the small amount of M. tuberculosis in a direct sample can result in test failures. In this experiment, M. tuberculosis identification and susceptibility prediction failed in 2/39 and 13/37 samples, respectively. MinION sequencing, in theory, allows sequencing to continue until sufficient coverage has been obtained, giving faster results when there is a high load and avoiding this type of failure when the load is low. The throughput obtained here with a 15% BCG-spiked sputum sample and R9.4 flow cells (1.3 Gb) would allow a turnaround time of 12.5 h (sample to complete results) or only 7.5 h to detection of M. tuberculosis and pyrazinamide resistance and placement on a phylogenetic tree.

We have predicted that identification to the species level and initial DST could be generated after 20 min (mean) of sequencing with MinION R9.4 and final DST within 150 min, providing the M. tuberculosis concentrations are sufficient (≥20% of the sequencing reads in this study). For these samples, it would be possible to multiplex sequencing and reduce per-sample costs. Conversely, for samples with low M. tuberculosis concentrations, nanopore sequencing would not provide sufficient data within 48 h. Although these predictions are based on a single R9.4 sequencing run, the data demonstrate clear scope for technology-driven improvement through improved mycobacterial enrichment and/or nonmycobacterial DNA depletion (Fig. 2a), higher sequencing yield, or real-time filtering of contamination (24).

Were this methodology implemented in clinical practice, we would expect a portion of direct sample to be retained for culture in all cases; this would be available for resequencing in case of insufficient sequencing depth and would allow distinction between live and dead bacilli. Unlike our study, where we had to use sample discards after clinical processing, if implemented, the increased sample volume could be used for sequencing closer to the time the sample was taken, presumably with greater success. Both Illumina and ONT technologies would require some level of sample batching, but for both, the turnaround time is likely to be much shorter than that of traditional phenotyping and could challenge same-day molecular tests such as Xpert MTB/RIF.

In conclusion, diagnostic and surveillance information can now be obtained directly from patient specimens in 16/44 h with the Illumina MiniSeq and MiSeq platforms, a considerable step forward. In addition, the ONT sequencing platform may offer the same information in as little as 7 to 12.5 h. Faster and more automated sample processing, as well as cost reductions, are clearly needed for adoption in low-income settings. Achieving this would revolutionize the management of TB.

## MATERIALS AND METHODS

### Sample selection and processing.

The ZN-positive direct respiratory samples with AFB scorings of +1 to +3 used in this study had been originally collected from patients with subsequently confirmed M. tuberculosis infections at the John Radcliffe Hospital, Oxford Universities NHS Foundation Trust, Oxford, United Kingdom (*n* = 18), and Birmingham Heartlands Hospital NHS Foundation Trust, Birmingham, United Kingdom (*n* = 22). Two of the 18 Oxford samples were culture-negative specimens taken 2.5 months apart from the same patient undergoing treatment for M. tuberculosis infection. If available, corresponding MGIT cultures were collected for each direct sample (Oxford, *n* = 11; Birmingham, *n* = 17). Two ZN- and culture-negative direct respiratory samples were also collected from the John Radcliffe Hospital.

The discarded direct samples were collected only after sufficient material had been obtained for the routine diagnostic work flow, including the requirement to ensure that enough sample volume remained if reculture was requested. Consequently, study samples were of lower volume and quality than would be the case if the method were used routinely. While waiting for the routine laboratory results, samples were stored at +4°C and later processed in batches of 5 to 12. All ZN-positive samples were digested and decontaminated with the NAC-PAC RED kit (AlphaTec, USA). Direct samples and corresponding MGIT culture aliquots (1 ml) were heat inactivated in a thermal block after sonication (20 min, 35 kHz) for 30 min and 2 h at 95C, respectively. MGITs were inactivated for 2 h owing to their heavy bacterial load. Before DNA extraction, samples were stored at +4°C.

### DNA extraction and Illumina MiSeq sequencing.

Mycobacterial DNA was extracted from MGIT cultures by a previously validated ethanol precipitation method (19). DNA was extracted from ZN-positive direct samples by a modified version of this protocol. These modifications included a saline wash followed by MolYsis Basic5 kit (Molzym, Germany) treatment for the removal of human DNA and addition of GlycoBlue coprecipitant (Life Technologies, USA) to the ethanol precipitation step (see Fig. S5).

Libraries were prepared for MiSeq Illumina sequencing by using a modified Illumina Nextera XT protocol (19). Samples were sequenced with the MiSeq Reagent kit v2, 2 × 150 bp in batches of 9 to 12 per flow cell. The median library size (TapeStation; Agilent, USA) was 627 bp (IQR, 495 to 681 bp). The median number of reads available per sample was 3.2 million (IQR, 2.8 to 4.1 million); this would yield a median depth of approximately 213, given pure M. tuberculosis, although in this study, we anticipated that nonmycobacterial reads would be present.

### DNA extraction for ONT MinION and Illumina MiniSeq sequencing.

ZN- and culture-negative sputum and BCG (Pasteur strain; cultivated at 37°C in MGIT tubes) DNA was extracted by a modified version of the method described in reference 19. Briefly, following a saline wash, samples were resuspended in 100 μl of molecular-grade water and subjected to three rounds of bead-beating at 6 m/s for 40 s. The beads were pelleted by centrifugation at 16,100 × *g* for 10 min, and 50 μl of supernatant was cleaned with 1.8 volumes of AMPure beads (Beckman Coulter, United Kingdom). Samples were eluted in 25 μl of molecular-grade water and quantified with a Qubit fluorimeter (Thermo Fisher Scientific, USA) (steps I, III, V, and VI of the MiSeq protocol [see Fig. S5]).

### MiniSeq sequencing.

Extracted ZN-negative sputum DNA and pure BCG DNA were combined in a 50:50 ratio (0.5 ng of each), and libraries were prepared alongside pure BCG DNA (1 ng) by using a modified Illumina Nextera XT protocol (19). BCG and two BCG-sputum DNA samples were sequenced at Illumina Cambridge Ltd., United Kingdom, with a Mid Output kit (FC-420-1004) reading 15 tiles and with 101 cycles.

### MinION sequencing.

All MinION sequencing utilized the best available sample preparation kit for our samples and flow cells (R9/R9.4 flow cells and PCR-based sample preparation, as described below). A single ZN-negative sputum extract was divided into three equal-concentration aliquots (187 ng), and BCG DNA was added at 5, 10, and 15% of the total sputum DNA concentration. These 5 to 15% spikes represent the lower end of the spectrum seen in the MiSeq samples described above (see Fig. 2a). These samples, along with pure BCG DNA, were prepared in accordance with ONTs PCR-based protocol for low-input libraries (DP006_revB_14Aug2015), with modified primers supplied by ONT, a 20-ng DNA input into the PCR, and LongAmp *Taq* 2× master mix (New England BioLabs, USA). The PCR conditions used were as follows: initial denaturation at 95°C for 3 min; 18 cycles of 95°C for 15 s, 62°C for 15 s, and 65°C for 2.5 min; and a final extension at 65°C for 5 min. Samples were cleaned in 0.4× volume AMPure beads, and the PCR product was assessed with a Qubit fluorimeter and TapeStation (Agilent, United Kingdom). The final elution was into 10 μl of 50 mM NaCl–10 mM Tris HCl, pH 8.0. Finally, 1 μl of PCR-Rapid Adapter (PCR-RAD; supplied by ONT) was added and samples were incubated for 5 min at room temperature to generate a presequencing mixture. The presequencing mixture was prepared for loading onto flow cells in accordance with standard ONT protocols, by using a loading amount of 50 to 100 fmol.

With the 15% BCG-spiked sputum DNA prepared as described above, amplification was repeated by using Phusion High-Fidelity PCR master mix with dimethyl sulfoxide (DMSO) (New England BioLabs, USA). Gradient PCR was performed to identify the optimal annealing temperature for recovery of BCG DNA (data not shown). The final PCR conditions were as follows: initial denaturation at 98°C for 30s; 18 cycles of 98°C for 10 s, 59°C for 15 s, and 72°C for 1.5 min; and a final extension of 72°C for 10 min. Following PCR, the sample was prepared for sequencing as described above. The final loading amount was approximately 27 fmol.

The above-described samples were sequenced with R9 spot-on generation flow cells and the 48-h protocol for FLO-MIN105 (ONT, United Kingdom). Base calling was performed via the Metrichor EPI2ME service (ONT, United Kingdom) with the 1D RNN for SQK-RAD001 v1.107 work flow.

Subsequently, a new 15% BCG-spiked sputum sample was prepared as described above, by using Phusion master mix with DMSO. Sequencing was performed with R9.4 spot-on generation flow cells and the 48-h FLO-MIN106 protocol (ONT, United Kingdom). The final loading amount was 43 fmol. Base calling was performed after sequencing was complete with Albacore (ONT, United Kingdom), as base calling via Metrichor failed. Subsequent tests of other samples (data not shown) showed that base calling could have been performed in real time during the run.

### Bioinformatic analysis of Illumina data.

To determine levels of contamination and M. tuberculosis in samples, reads were immediately mapped with bwa_mem (25) to the human reference genome GRCh37 (hg19) and human reads were counted and permanently discarded. The remaining stored reads were then mapped to the M. tuberculosis H37Rv reference strain (GenBank NC_018143.2), and any unmapped reads were then mapped to nasal, oral, and mouth flora available in the NIH Human Microbiome Project database (http://www.hmpdacc.org/).

Mycobacterial species and antibiotic resistance to isoniazid, rifampin, ethambutol, pyrazinamide, streptomycin, aminoglycosides (including capreomycin, amikacin, and kanamycin), and fluoroquinolones (including moxifloxacin, ofloxacin, and ciprofloxacin) was predicted with Mykrobe predictor software (26) v0.3.5, updated with a new validated catalogue of resistance-conferring genetic mutations (see Table S3; from reference 1). For samples where the estimated depth of k-mer coverage of M. tuberculosis reported by Mykrobe predictor fell below 3×, no resistance predictions were made. The precise command used was mykrobe predict SAMPLE_ID tb -1 FASTQ –panel walker-2015 –min-depth 3.

### Phylogenetic analysis of pairs.

Conservative SNP calls were made with Cortex (27) (independent work flow, k = 31) on 3,480 samples from reference 1. Singleton variants were discarded, and a deduplicated list of 68,695 SNPs was constructed. All samples (from our study and from reference 1) were genotyped at these sites by using the Mykrobe predictor genotyping model (26). All 27 of the MGIT samples had high coverage, but several of the direct samples had low coverage (Fig. 3a, bottom left). For this comparison, we excluded pairs where the direct sample had <5× coverage to ensure like-with-like analysis, leaving 17 pairs. We then measured the number of SNP differences between the paired direct and MGIT samples, counting only sites where both genotypes had high confidence in our Illumina model (difference between log likelihood of called genotype [e.g., ALT allele] and log likelihood of uncalled genotype [e.g., REF allele] of >1), and neither site was called as heterozygous.

Samples were placed on the phylogenetic tree of 3,480 samples from reference 1 by identifying the leaf with the fewest SNP differences across the 68,695 sites. Placement therefore returns a closest leaf and a SNP distance to that leaf.

### Statistical analysis.

Univariable and multivariable linear regression was used to identify independent factors affecting the log_10_ DNA concentration after extraction. Analyses were performed with Stata 14.1 (2015; StataCorp, USA).

### Bioinformatic analysis of MinION data.

Mykrobe predictor version v0.5.0-6-g6b19d83 was used to predict resistance from the MinION base called reads (command: mykrobe predict SAMPLE_ID tb −1 FASTQ –panel walker-2015 –ont). This uses an ONT-specific genotyping model, a modification of that published in reference 26. Specifically, it uses a Poisson model of total k-mer counts on alternate alleles (instead of using the median k-mer coverage) and applies a “genotype confidence” threshold of 100 (difference between log likelihood of called genotype (e.g., ALT allele) and that of uncalled genotype (e.g., REF allele) of >100). Figures S6 and S7 show the genotype confidence distribution split by whether the genotype is correct or not.

Yield and timing were analyzed with Poretools (28). For the R9.4 sample, Mykrobe predictor was applied to the cumulative read output at each hour. The yield of BCG was measured by mapping to a BCG reference (accession no. BX248333.1).

Phylogenetic placement of the 15% spike BCG sample sequenced on MinION R9.4 was achieved as for the Illumina data—by genotyping 68,695 SNPs and choosing the leaf with the fewest SNP differences across those sites.

### MinION error analysis.

Error bias in the consensus of MinION R9 1D pure BCG reads was measured in two ways by using reads from the pure BCG sequencing run described above. By method 1, reads were mapped to the M. tuberculosis reference genome by using bwa_mem, and then this was passed to the consensus tool racon (29). The output of this was compared with the BCG reference genome with MUMMER (30). Since we were comparing M. bovis BCG with its own reference genome, any observed SNPs were due either to sequencing errors or to evolution since the reference genome was constructed. We assumed that the latter were negligible in comparison with the error rate in nanopore reads (see Fig. S4) and considered all SNPs to be errors. Bias in these errors was observed by looking at isolated SNPs (avoiding alignment artifacts due to nearby indels). The results are shown in Table S4. By method 2, *de novo* assembly was performed with Canu (31), and then this was compared with the BCG reference genome with MUMMER as described above. The results are shown in Table S5.

The mapping approach (method 1 described above) found that 28% of the consensus errors were A to G and 60% were T to C. (Note that these refer to the SNP with respect to the reference and not to errors within a single read passing through a pore.) The *de novo* assembly approach found that 50% of the consensus errors were A to G and 44% were T to C. Although the estimates differed quantitatively, they agreed on the existence and direction of the bias.

### MinION turnaround estimates obtained with empirical M. tuberculosis read proportion data.

The proportion of M. tuberculosis reads found in our clinical samples varied over a considerable range (Fig. 2a, blue), with 0.3 to 97.9% of the sequenced DNA coming from M. tuberculosis. To model how this distribution might translate into MinION performance, we used hourly time stamps on the R9.4 MinION total DNA yield curve (see Fig. S3) and coverage needed to detect M. tuberculosis, pyrazinamide resistance, and full susceptibility results to estimate the turnaround times for all Illumina-sequenced samples, supposing that they all were to yield 1.3 Gb of MinION reads with the same proportion of reads from M. tuberculosis as seen in Fig. 2a. The results are displayed in Fig. S8 and Table S6, with samples ordered by increasing proportions of M. tuberculosis.

### Costing analysis.

Basic costing included reagents required for sample decontamination, DNA extraction, MiSeq and Nanopore library preparation, and sequencing (correct as of November 2016). Generic laboratory consumables (e.g., pipette tips, tubes) were not included. SureSelect (Agilent, United Kingdom) costs, as used by Brown et al. (18), were obtained via a company representative and were correct of June 2016. U.S. dollars were converted to Great British pounds (GBP) at $1.25/GBP. See Table S7 for details.

### Ethics.

For this study, no ethical review was required because it was a laboratory method development study focusing on bacterial DNA extracted from discarded samples identified only by laboratory numbers with no personal or clinical data. Sequencing reads identified as human on the basis of fast mapping with BWA were counted and immediately permanently discarded (i.e., never stored electronically).

### Accession number(s).

The MiSeq, MiniSeq, and MinION data obtained in this study have been deposited in the NCBI Sequence Read Archive under study accession number SRP093599.

## Supplementary Material

Supplemental material
