# Supplementary material for: Same-Day Diagnostic and Surveillance Data for Tuberculosis via Whole-Genome Sequencing of Direct Respiratory Samples
Source: J Clin Microbiol. 2017 Apr 25;55(5):1285–98. doi: 10.1128/JCM.02483-16 (PMC5405248; doi:10.1128/JCM.02483-16)
Supplement: Supplemental material [file supp_55_5_1285__index.html]

Same-Day Diagnostic and Surveillance Data for Tuberculosis via Whole-Genome Sequencing of Direct Respiratory Samples — Supplemental material 

# Same-Day Diagnostic and Surveillance Data for Tuberculosis via Whole-Genome Sequencing of Direct Respiratory Samples

## Supplemental material

- Supplemental file 1 -

  Fig. S1 (Comparison of within-sample diversity in paired direct and MGIT samples), S2 (MinION R9 read coverage across BCG reference genome), S3 (Cumulative yield from MinION R9), S4 (MinION R9 1D read identity distribution), S5 (Direct respiratory sample DNA extraction protocol for Illumina sequencing), S6 (Genotype confidence [frequency] distribution for correctly/incorrectly genotyped SNPs [R9.4 MinION]), S7 (Genotype confidence [density] distribution for correctly/incorrectly genotyped SNPs [R9.4 MinION]), and S8 (Predicted performance of MinION R9.4 using empirical distribution of proportion of *M. tuberculosis* [plot]) and Tables S1 (Reference lab susceptibility results and WGS-based predictions for Illumina-sequenced samples), S2 (Resistance mutations identified in direct and MGIT samples), S3 (Resistance catalog used by Mykrobe predictor), S4 (Consensus error biases in R9 MinION data [estimated using mapping]), S5 (Consensus error biases in R9 MinION data [estimated using assembly]), S6 (Predicted performance of MinION R9.4 using empirical distribution of proportion of *M. tuberculosis* [data]), and S7 (Pricing estimates for consumables)

  PDF, 1.3M
