# Supplementary material for: Same-Day Diagnostic and Surveillance Data for Tuberculosis via Whole-Genome Sequencing of Direct Respiratory Samples
Source: J Clin Microbiol. 2017 Apr 25;55(5):1285–98. doi: 10.1128/JCM.02483-16 (PMC5405248; doi:10.1128/JCM.02483-16)
Supplement: Supplemental material [file JCM.02483-16_zjm999095477s1.pdf]

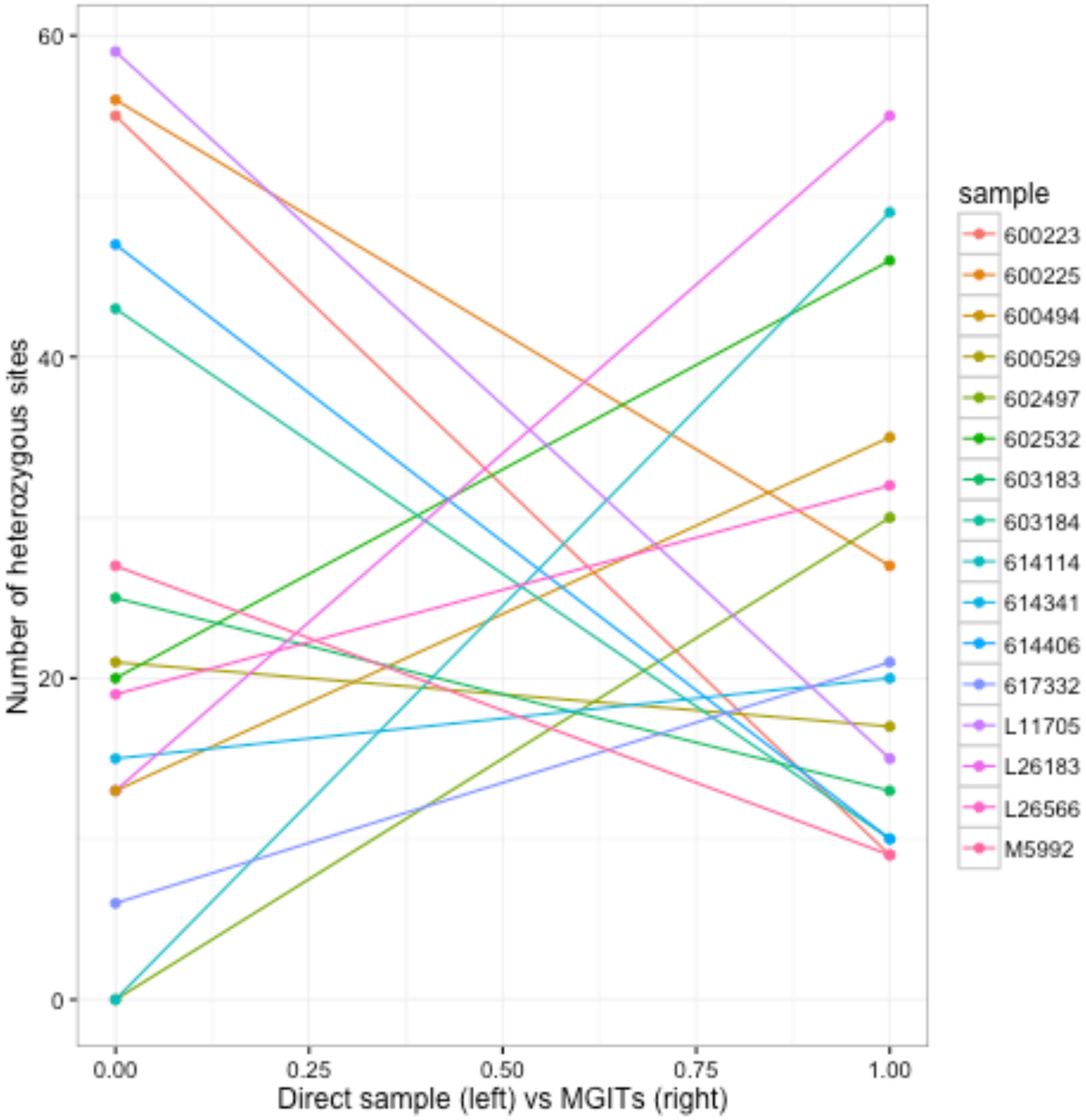

Supplementary Figure 1: Number of confident (here, for Illumina model this means genotype confidence>1) heterozygous SNPs called in paired vs direct/MGIT samples where both samples have at least 5x depth of coverage. There was no consistent pattern of more heterozygotes in the direct samples.

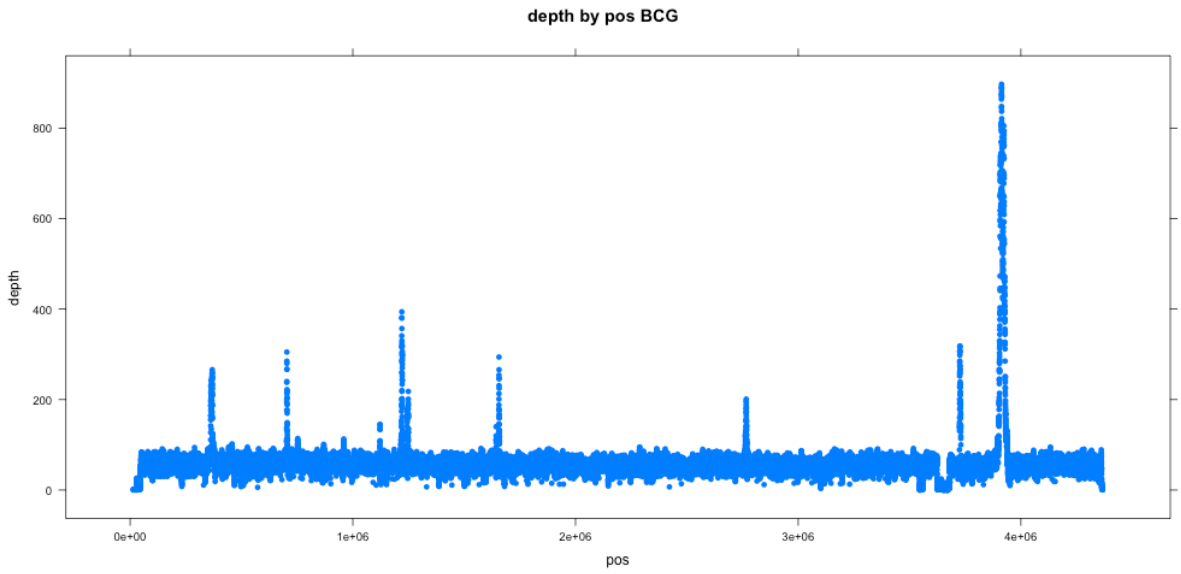

Supplementary Figure 2:

Coverage distribution across *M. bovis* BCG strain reference genome (from ftp://ftp.sanger.ac.uk/pub/project/pathogens/Mycobacterium/bovis) of MinION reads from pure *M. bovis* BCG strain culture, showing even coverage across the genome apart from 7 loci with peaks of coverage.

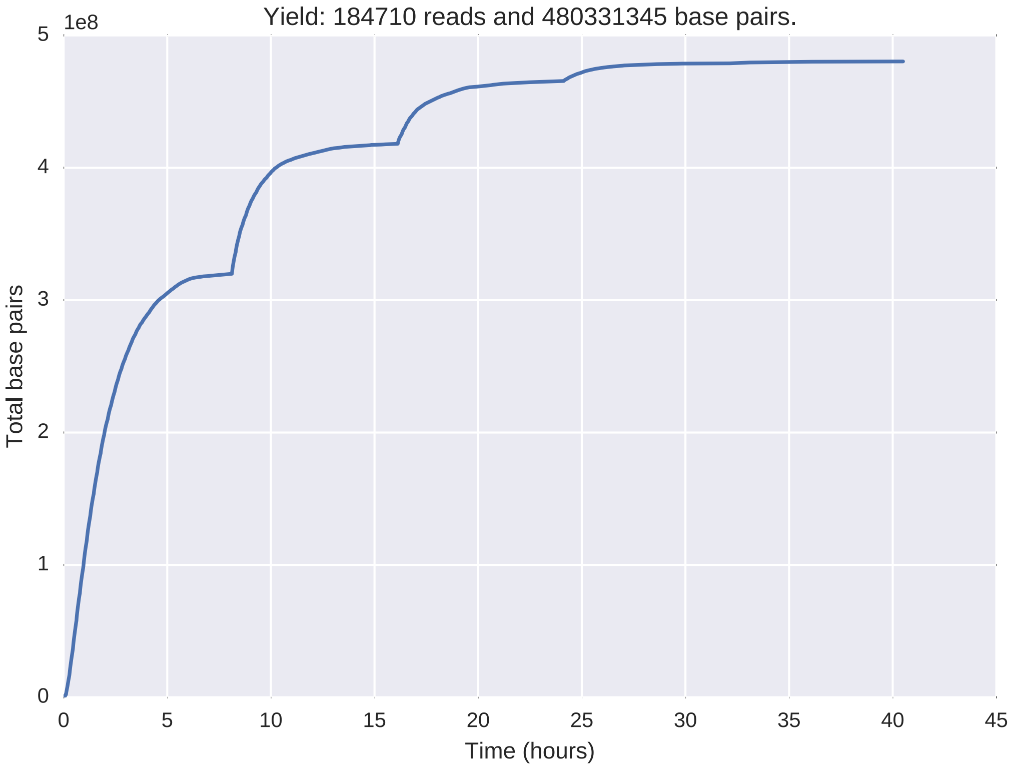

Supplementary Figure 3: Cumulative yield (in megabases) from the MinION (version R9 flow cell) when sequencing culture-negative sputum spiked with 15% BCG, using the Phusion PCR enzyme. Approximately 65% of the final yield is obtained in 8 hours and 80% in 10 hours.

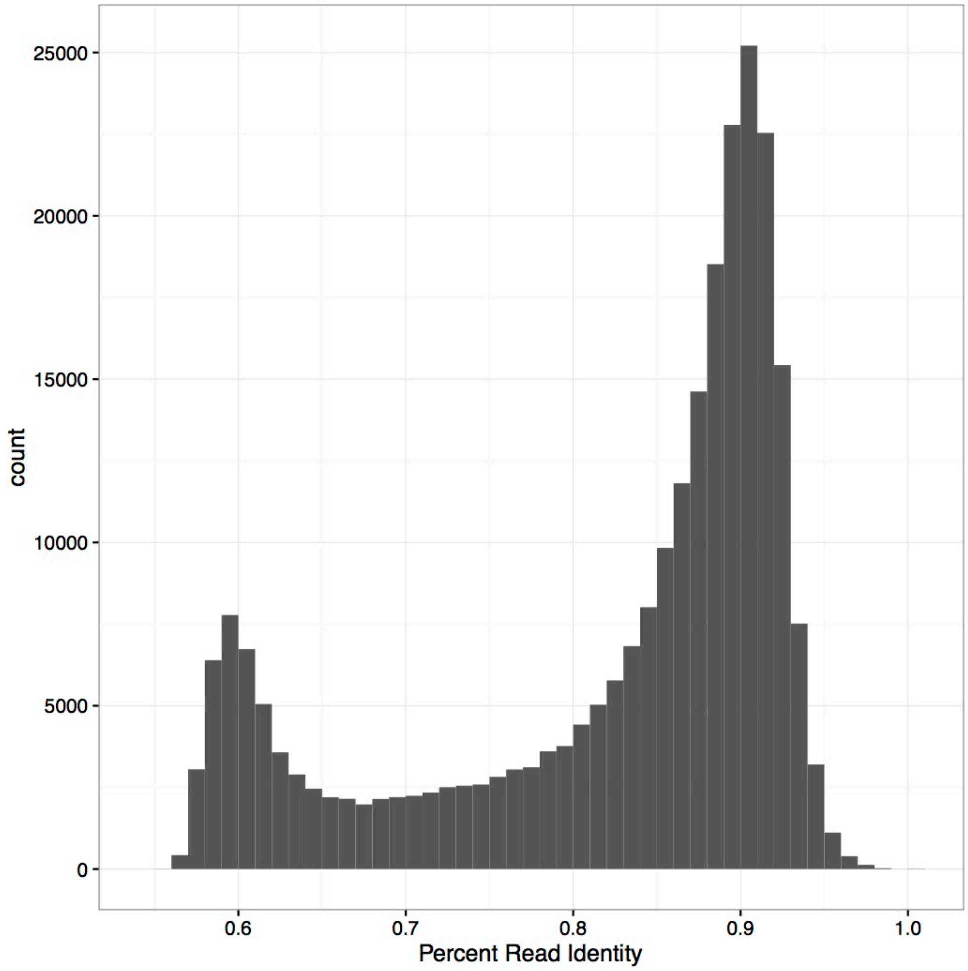

Supplementary Figure 4: Identity distribution for 1D reads in pure BCG sequencing run with MinION (version R9 flow cell). Each read is mapped to the BCG reference genome and percentage identity measured, and then the distribution of these scores is shown

DNA extraction protocol from the direct respiratory samples

I. Saline wash

a. Centrifuge 1ml of heat-inactivated sample for 15 min at 13,000 rpm
b. Remove as much supernatant as possible by pipetting without disturbing a pellet
c. Add 1 ml of sterile phosphate buffered saline (PBS) and re-suspend pellet by pipetting
d. Optional: repeat steps (a-c)

II. Removal of human DNA with MolYsis Basic5 kit

a. Add 250 ul of buffer CM to 1 ml of sample
b. Incubate at room temperature for 5 min
c. Add 250 ul buffer DB and 10 ul MolDNase B (do not premix) to each sample and immediately vortex for 10s
d. Incubate at room temperature for 15 min
e. Centrifuge for 10 min at 13,000 rpm
f. Remove supernatant by pipetting without disturbing a pellet
g. Add 1ml of buffer RS and pipette up and down until the pellet is fully re-suspended
h. Centrifuge for 10 min at 13,000 rpm
i. Remove supernatant by pipetting without disturbing the pellet
j. Add 700 ul of sterile distilled water and re-suspend the pellet by pipetting up and down

III. Mechanical cell disruption

a. Transfer 700 ul of sample to a 2 ml lysing matrix B tube (MB Biomedicals, USA)
b. Bead beat in FastPrep-24 tissue homogeniser (or any other homogenizer) 3 times for 40 s at 6.0 m/s
c. Centrifuge for 10 min at 13,000 rpm
d. Transfer 450 ul of supernatant to new 1.5 ml tube

IV. Ethanol precipitation

a. Add 1/10 volume (45 ul) of 3M sodium acetate to 450 ul of supernatant
b. Add 1.5 ul of GlykoBlue Coprecipitant (Life Technologies, USA)
c. Add 2 volumes (1 ml) of ice-cold 96% EtOH and vortex for 10 s
d. Incubate at -20°C for 30 min to 1 h
e. Centrifuge at 13,000 rpm for 15 min and remove supernatant by pipetting
f. Add 1 ml of 70% EtOH, incubate for 1 min and remove as much supernatant as possible without disturbing the pellet
g. Dry at room temperature for 10-15 min (avoid to over-dry the pellet)
h. Re-suspend the pellet in 50 ul of TE (Tris-EDTA, pH 8.0) buffer by heating at 50-55°C for 10 min. Vortex samples 2-3 times during the incubation. Large pellet might be still visible at the end of incubation
i. Transfer 45-50 ul of supernatant from the tubes to the 96 well plate

V. DNA clean-up with AMPure XP magnetic beads

a. Add x1.8 volume of beads to 45-50 ul of DNA
b. Seal the plate and mix well by vortexing for 30 s
c. Incubate plate for 10 min at room temperature, spin the plate briefly
d. Place plate on the magnetic holder for 3 min and remove the supernatant by pipetting without disturbing the beads
e. Leave the plate on a magnetic holder, add 200 ul of 80% EtOH, incubate for 1 min and remove EtOH by pipetting
f. Repeat step (e) one more time
g. Air-dry beads at room temperature for 10 min after the second wash
h. Add 26 ul of buffer TE and pipette up and down until beads are completely re-suspended
i. Incubate plate at room temperature for 5 min
j. Place plate on the magnetic holder for 3 minutes
k. Transfer 25 ul of supernatant without beads to the new tubes\plate

VI. Quality Control

a. Measure DNA concentration using Qubit Fluorometer (Life Technologies, USA)
b. Store ready DNA at -20°C

Supplementary Figure 5. DNA extraction protocol from the direct respiratory samples as used for Illumina sequencing. Modification of this protocol for MinION is described in main text.

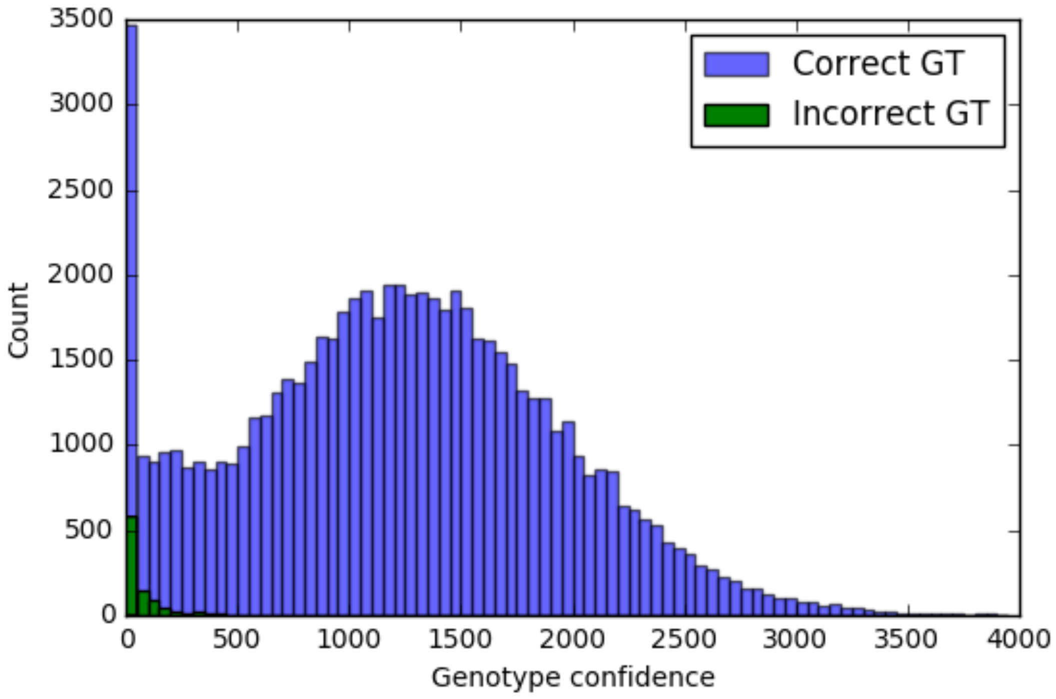

Supplementary Figure 6. Distribution of "genotype confidence" across 68950 SNPs genotyped on R9.4 MinION reads. To clarify, if under our ONT-model, the log likelihood of a particular A/G SNP having allele A is (say) -0.01, and the log likelihood of the allele being G is -1000, then the SNP is genotyped as having allele A with confidence -0.01-(-1000)= 999.99. Larger values of genotype confidence signify more certain results. Here, bars are split by whether the genotype was correct (blue) or incorrect (green) (using MiniSeq genotypes as truth). Mykrobe predictor uses a genotype confidence threshold of 100 for ONT genotyping.

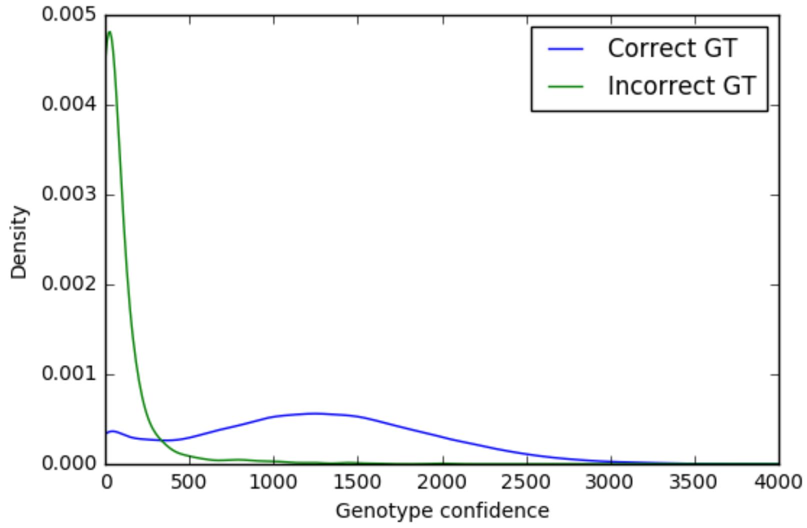

Supplementary Figure 7. As Supp. Fig.6 but plotting density rather than counts.

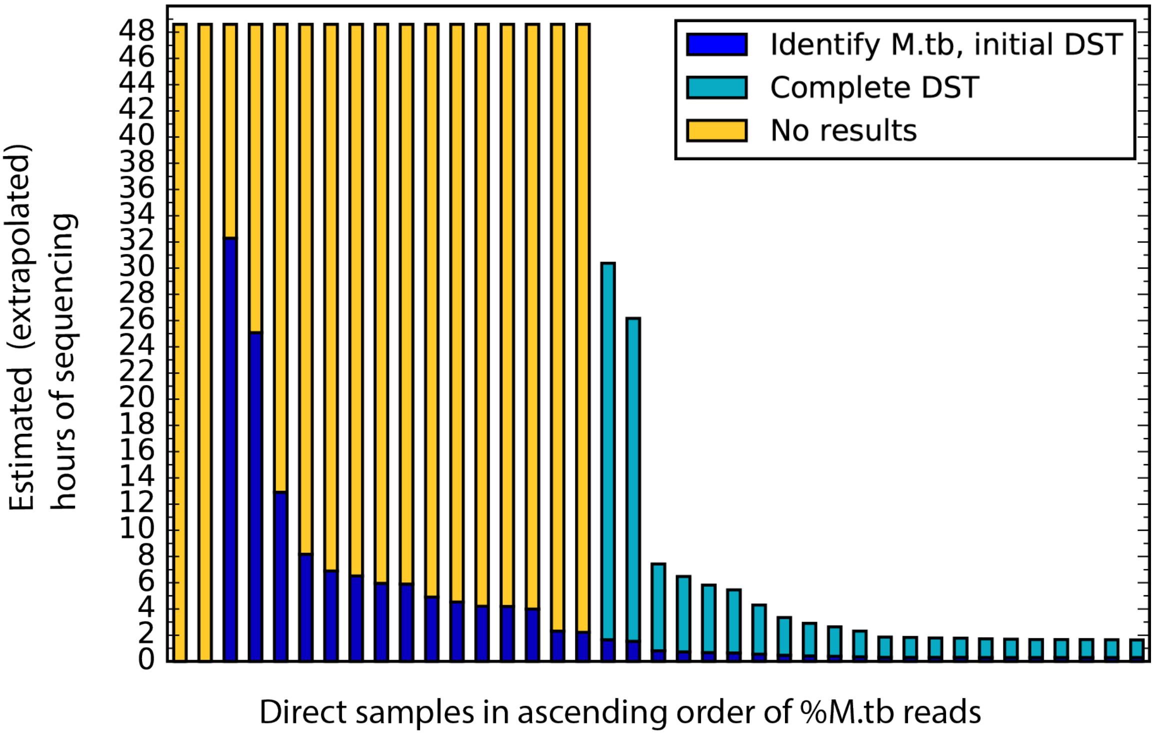

Supplementary Figure 8

Extrapolating from MinION R9.4 results to estimate performance for a realistic distribution of proportion of reads from *M. tuberculosis.* Each bar corresponds to one of the 39 samples sequenced with Illumina MiSeq sorted by increasing proportion of *M. tuberculosis*. We assume that, if sequenced with a R9.4 MinION, each of the 39 samples would yield as much as our R9.4 run (1.3Gb), and infer the yield of *M. tuberculosis* reads for each sample from the proportion (blue) shown in Figure 2a. This gives us estimates of sequencing time needed to detect *M. tuberculosis* (dark blue), and provide susceptibility predictions (light blue, “abbreviated” as DST in legend). We mark in yellow time spent sequencing without attaining a result. Yield from MinION flow cells is variable and has changed considerably within the course of this study, but for a fixed yield, this plot gives an idea of how sequencing time and success vary with the proportion of reads coming from *M. tuberculosis*.

Supplementary Table 1. Antibiotic susceptibility predictions by reference laboratory from MGIT cultures and by WGS directly from clinical samples (n=37), MGIT cultures

Sample ID	Reference laboratory DST							WGS direct samples							WGS MGIT culture						
	Isoniazid	Rifampicin	Ethambutol	Pyrazinamide	Streptomycin	Aminoglycosides	Fluoroquinolones	Isoniazid	Rifampicin	Ethambutol	Pyrazinamide	Streptomycin	Aminoglycosides	Fluoroquinolones	Isoniazid	Rifampicin	Ethambutol	Pyrazinamide	Streptomycin	Aminoglycosides	Fluoroquinolones
F13757	S	S	S	S	.	.	.	F	F	F	F	F	F	F	.	.	.	.	.	.	.
M5979	S	S	S	S	.	.	.	F	F	F	F	F	F	F	S	S	S	S	S	S	S
M5992	S	S	S	S	.	.	.	S	S	S	S	S	S	S	S	S	S	S	S	S	S
L48766	S	S	S	S	.	.	.	F	F	F	F	F	F	F	S	S	S	S	S	S	S
H32593	S	S	S	S	.	.	.	F	F	F	F	F	F	F	.	.	.	.	.	.	.
L11705	S	S	S	S	.	.	.	S	S	S	S	S	S	S	S	S	S	S	S	S	S
F33033	R	S	S	S	.	.	.	F	F	F	F	F	F	F	.	.	.	.	.	.	.
600223	S	S	S	S	.	.	.	S	S	S	S	S	S	S	S	S	S	S	S	S	S
600225	S	S	S	S	.	.	.	S	S	S	S	S	S	S	S	S	S	S	S	S	S
600494	S	S	S	S	.	.	.	S	S	S	S	S	S	S	S	S	S	S	S	S	S
600529	S	S	S	S	.	.	.	S	S	S	S	S	S	S	S	S	S	S	S	S	S
602112	R	S	R	S	.	.	.	R	R	R	M	S	M	R	.	.	.	.	.	.	.
602188	S	S	S	S	.	.	.	S	S	S	S	S	S	S	.	.	.	.	.	.	.
602378	S	S	S	S	.	.	.	S	S	S	S	S	S	S	.	.	.	.	.	.	.
602497	S	S	S	S	.	.	.	S	S	S	S	S	M	S	S	S	S	S	S	S	S
602532	R	S	S	S	.	.	.	R	S	S	S	S	S	S	R	S	S	S	S	S	S
602861	S	S	S	S	.	.	.	F	F	F	F	F	F	F	S	S	S	S	S	S	S
602905	R	R	R	S	.	.	.	R	R	R	M	S	M	R	.	.	.	.	.	.	.
602907	R	R	S	S	.	.	.	F	F	F	F	F	F	F	R	R	S	S	S	S	S
602908	R	R	S	S	.	.	.	F	F	F	F	F	F	F	R	R	S	S	S	S	S
603183	R	S	S	S	.	.	.	R	S	S	S	S	S	S	R	S	S	S	S	S	S
603184	R	S	S	S	.	.	.	R	S	S	S	S	S	S	R	S	S	S	S	S	S
614114	R	S	S	S	.	.	.	R	S	S	S	S	M	S	R	S	S	S	S	S	S
614115	R	S	S	S	.	.	.	R	S	S	S	S	M	S	R	S	S	S	S	S	S
614341	S	S	S	S	.	.	.	S	S	S	S	S	S	S	S	S	S	S	S	S	S
614406	S	S	S	S	.	.	.	S	S	S	S	S	S	S	S	S	S	S	S	S	S
614509	R	R	R	R	.	.	.	R	R	R	M	S	M	R	.	.	.	.	.	.	.
617126	S	S	S	S	.	.	.	F	F	F	F	F	F	F	S	S	S	S	S	S	S
617332	S	S	S	S	.	.	.	S	S	S	S	S	S	S	S	S	S	S	S	S	S
L26916d	S	S	S	S	.	.	.	S	S	S	S	S	S	S	.	.	.	.	.	.	.
H54502	R	S	S	S	.	.	.	F	F	F	F	F	F	F	R	S	S	S	S	S	S
L26183	S	S	S	S	.	.	.	S	S	S	S	S	S	S	S	S	S	S	S	S	S
L26556	S	S	S	S	.	.	.	S	S	S	S	S	S	S	S	S	S	S	S	S	S
L26916	S	S	S	S	.	.	.	S	S	S	S	S	S	S	.	.	.	.	.	.	.
L61938	S	S	S	S	.	.	.	F	F	F	F	F	F	F	S	S	S	S	S	S	S
L66375	R	R	R	R	.	.	.	F	F	F	F	F	F	F	R	R	R	R	S	S	S
M26794	S	S	S	S	.	.	.	F	F	F	F	F	F	F	S	S	S	S	S	S	S

S= sensitive, R=resistant, M= mixed result (presence of S and R variants), F=failed for the reference laboratory DST /falls below Mykrobe confidence threshold for WGS, .=not tested.

Supplementary Table 2. Antibiotic resistance conferring mutations identified by WGS in direct samples and if available corresponding MGIT cultures. [1]m after sample ID indicates MGIT culture; [2]resistance conferring mutation in the relevant gene, R:S:C represents the number of reads on the R allele, the number of reads on the S allele and C represents Mykrobe Illumina-model confidence score (this is difference between log likelihoods of most and next-most likely models, so any value greater than 1 is confident); (R = resistant, S= sensitive)

Sample ID[1]	Patient	Mutation_R:S:C[2]					
		Isoniazid	Rifampicin	Ethambutol	Pyrazinamide	Aminoglycosides	Fluoroquinolones
614114	1	katG_S315T_21:0:99				rrs_C1402A_19:22:43	
614114m	1	katG_S315T_78:0:99					
614115	1	katG_S315T_48:2:69				rrs_C1402A_55:59:124	
614115m	1	katG_S315T_85:0:99					
614509	2	fabG1_C15T_35:0:99	rpoB_I491F_27:0:99	embB_M306I_30:1:44	pncA_V7L_17:13:23	rrs_A1401G_19:39:39	gyrA_D94G_31:0:99
602112	2	fabG1_C-15T_35:4:40<br>katG_S315T_38:44:85	rpoB_I491F_50:1:76	embB_M306I_34:0:99	pncA_V7L_27:9:8<br>pncA_T135P_11:37:20	rrs_A1401G_36:42:81	gyrA_D94G_41:0:99
602905	2	fabG1_C-15T_130:0:99	rpoB_I491F_168:0 :99	embB_M306I_181:0:99	pncA_V7L_119:57:32	rrs_A1401G_73:91:164	gyrA_D94G_156:0:99
602532	3	fabG1_C-15T_174:0:99					
602532m	3	fabG1_C-15T_98:1:152					
603183	4	fabG1_C-15T_155:0:99					
603183m	4	fabG1_C-15T_33:0:99					
603184	4	fabG1_C-15T_129:0:99					
603184m	4	fabG1_C-15T_84:2:126					

Supplementary Table 3

Table of 175 amino acid or DNA mutations included in the Mykrobe predictor panel. Notation is either 1) aNNb, where a is susceptible amino acid allele, and b is resistant amino acid allele, and NN is the position of the amino acid in the gene, or 2) a-Nb, where a is a nucleotide, the mutation occurs N bases before the start of the gene, and b is the resistant allele. X refers to any other allele. A/B means A or B.

Drug	Mutations
Amikacin	rrs_A1401X,rrs_C1402X,rrs_G1484X
Capreomycin	gid_Y195H,rrs_A1401X,rrs_C1402X,rrs_G1484X,tlyA_C-83T
Ethambutol	embA_C-12T,embA_C-16G/T,embB_D328Y,embB_D354A,embB_G406A/D/S,embB_M306X,embB_N1033K,embB_Q497K/R
Isoniazid	ahpC_C-57T,ahpC_C-72T,fabG1_A-16X,fabG1_C-15X,fabG1_G-17X,fabG1_T-8X,inhA_I194T,inhA_I21T,inhA_S94A,katG_A109V,katG_A614E,katG_D142G,katG_G125D,katG_G182R,katG_G297V,katG_L141F, katG_L159P,katG_L627P,katG_L704S,katG_P232R,katG_R104Q,katG_S315X,katG_S700P,katG_T180K,katG_V633A,katG_W191G/R, katG_W300C/S,katG_W328L,katG_W505X,katG_W90R,ndh_G225D
Kanamycin	eis_C-10T,rrs_A1401X,rrs_C1402X,rrs_G1484X
Pyrazinamide	pncA_A-12G,,pncA_C138R,pncA_C14R,pncA_D12A,pncA_D136N,pncA_D49N,pncA_D8G/N,pncA_F81V,pncA_G132D,pncA_G162D, pncA_G78C,pncA_G97C/D/R,pncA_H137R,pncA_H51Q/D,pncA_H57R,pncA_H71Q/Y,pncA_I133T,pncA_K48E,pncA_K96T, pncA_L151S,pncA_L159V,pncA_L172P,pncA_L27P,pncA_L4S,pncA_M175V,pncA_P54L/Q,pncA_Q10X,pncA_Q141X,pncA_S104G/R, pncA_S32I,pncA_T-11C,pncA_T114P,pncA_T135P,pncA_T47A,pncA_V125G,pncA_V139L,pncA_V180F/G,pncA_V21G,pncA_V7L, pncA_W68C/R,pncA_Y99X
Quinolones	gyrA_A74S,gyrA_A90X,gyrA_D89X,gyrA_D94X,gyrA_G88X,gyrA_H85X,gyrA_H87X,gyrA_I92X,gyrA_P86X,gyrA_S91X,gyrA_Y93X
Rifampicin	rpoB_A451X,rpoB_D435X,rpoB_F425X,rpoB_F433X,rpoB_G426X,rpoB_G442X,rpoB_G981D,rpoB_H445X,rpoB_I491F, rpoB_K446X,rpoB_L430X,rpoB_L440X,rpoB_L443X,rpoB_L452X,rpoB_M434X,rpoB_N437X,rpoB_N438X,rpoB_P439X, rpoB_Q429X,rpoB_Q432X,rpoB_Q436X,rpoB_R447X,rpoB_R448X,rpoB_S428X,rpoB_S431X,rpoB_S441X,rpoB_S450X,

	rpoB_T427X,rpoB_T444X,rpoB_T676P,rpoB_V170F,rpoB_V359A,rpsA_E67D,rpsA_V260I
Streptomycin	gid_A134E,gid_A138T/V,gid_A19P,gid_A200E,gid_A205E,gid_A80P,gid_C52F,gid_D85A,gid_E173X,gid_G117V,gid_G30D, gid_G34V,gid_G69D,gid_G73A,gid_H48N/Q,gid_I11N,gid_I162S,gid_L26F,gid_L79S/W,gid_L91P,gid_P75L/R,gid_P93L, gid_Q125X,gid_R118L/S,gid_R137P/W,gid_R47W,gid_R64W,gid_R83P,gid_S136X,gid_S149R,gid_S70N,gid_V203L,gid_V41I, gid_V65G,gid_V88A,rpsL_K43R,rpsL_K88R,rrs_A1325C,rrs_A514X,rrs_C513X,rrs_C516X,rrs_C517X,rrs_C905A,rrs_G515X

Supplementary Table 4

Consensus error biases within BCG MinION data, measured on racon consensus of reads mapped to *M. tuberculosis* reference genome. Statistics taken from the 1776 error SNPs which were isolated (at least 10bp from any other variant) in order to avoid indel alignment issues.

Error SNP	Count	Proportion (%)
A->T	3	0.2
A->C	11	0.6
A->G	497	28.0
T->A	5	0.3
T->C	1072	60.4
T->G	41	2.3
C->A	4	0.2
C->T	43	2.4
C->G	10	0.6
G->A	79	4.5
G->T	5	0.3
G->C	6	0.3

Supplementary Table 5

Consensus error biases within BCG MinION data, measured on a Canu *de novo* assembly of same BCG dataset as in Supplementary Table 4. Statistics taken from the 861 error SNPs which were isolated (at least 10bp from any other variant) in order to avoid indel alignment issues.

Error SNP	Count	Proportion (%)
A->T	0	0
A->C	8	0.9
A->G	428	49.7
T->A	1	0.1
T->C	375	43.6
T->G	5	0.6
C->A	0	0
C->T	19	2.2
C->G	2	0.2
G->A	23	2.7
G->T	0	0
G->C	0	0

Supplementary Table 6

Prediction of time taken to identify *M. tuberculosis* (and provide initial susceptibility predictions) and of time taken to give final susceptibility predictions with MinION R9.4. for a range of different proportions of *M. tuberculosis.* Estimates are based on the proportion of reads that mapped to the *M. tuberculosis* reference genome (column 1), from each of the 39 samples sequenced with Illumina MiSeq. Each sample is assumed to generate 1.3Gigabases of reads, arriving according to the yield curve from our R9.4 run (Supplementary Figure 3). From these, and the depth of coverage at the hourly timestamps at which our R9.4 run detected Mtb/produced final predictions, one can predict the equivalent time for all 39 samples. If the result is not achieved within 48 hours, we mark it as N/A. We highlight two pairs of statistics. First, for samples with >=20% reads from *M. tuberculosis*, identification of *M. tuberculosis* would take 20 minutes, and complete results would take 150 minutes. Second, for samples with >=84% reads from *M. tuberculosis*, identification of *M. tuberculosis* would also take 20 minutes, but full results would take 93 minutes.

% of reads mapping to *M. tuberculosis*	Identify Mtb+initial susceptibility predictions (hours)	Complete susceptibility predictions (hours)
0.28	N/A	N/A
0.64	N/A	N/A
0.83	32.28	N/A
0.95	25.08	N/A
1.23	12.90	N/A
1.83	8.16	N/A
2.06	6.89	N/A
2.14	6.51	N/A
2.30	5.95	N/A
2.32	5.89	N/A
2.69	4.90	N/A

2.88	4.53	N/A
3.07	4.20	N/A
3.09	4.18	N/A
3.24	3.98	N/A
5.67	2.31	N/A
5.91	2.21	N/A
8.23	1.65	28.72
9.07	1.52	24.64
19.99	0.80	6.63
22.30	0.72	5.75
24.42	0.67	5.14
25.91	0.64	4.80
32.30	0.54	3.75
41.86	0.46	2.88
49.19	0.41	2.48
54.89	0.38	2.25
64.35	0.35	1.95
84.01	0.30	1.55
86.04	0.30	1.52
89.13	0.30	1.48
89.40	0.30	1.47
92.90	0.29	1.42
94.78	0.29	1.39
95.86	0.29	1.37
96.18	0.29	1.37
96.50	0.29	1.36
96.79	0.29	1.36
97.89	0.28	1.34

Supplementary Table 7:

MinION pricing below assumes 1 sample per flow cell.

Procedure	Cost per sample (GBP)	Workflow(s)
DNA extraction	19.58	MiSeq, MiniSeq, MinION, SureSelect
Nextera XT sample preparation	26.34	MiSeq, MiniSeq
MiSeq Reagent Kit v2, 2 x 150bp (12 samples per kit)	49.63	MiSeq, SureSelect
MiniSeq Mid Output kit (3 samples per kit)	152.53	MiniSeq
SureSelect (5.9Mb kit) sample preparation including sequencing library preparation	153.60	SureSelect
MinION sample preparation	95.31	MinION
MinION flow cell	400.00	MinION
**TOTAL WORKFLOW REAGENT COSTS**		
MiSeq	95.55	
MiniSeq	198.45	
SureSelect	203.23	
MinION	514.89	
